# The Season: Its Diseases and Their Prevention

**Published:** 1855-12

**Authors:** Benjamin W. Richardson

**Affiliations:** Physician to the Royal Infirmary for Diseases of the Chest.


### THE SEASON: ITS DISEASES AND THEIR PREVENTION.

By Benjamin W. Richardson, M.D., Physician to the Royal Infirmary for Diseases of the Chest.

The distinguished Arbuthnot laid it down as an aphorism, that every season has its special diseases. This is in some measure true; but as the seasons of one year vary very much from the seasons of another year, and as the division into seasons is, after all, arbitrary, the rule must be received with many exceptions.

The prevalence of diseases as a whole, and of the mortality arising from them, is, however, well marked in various periods of the year. I made a careful analysis of the facts bearing on this subject two years ago, and obtained very important results. The analysis refers only to the diseases of certain parts of England, and is made from mortality tables; viz., those published by the Registrar General; but from its wide basis, its results give a fair picture of the special season diseases of England.

The analysis includes deductions made from not fewer than 139,318 deaths, occurring during years, extending from 1838 to 1853, and arising from the following diseases: small-pox, measles, scarlet fever, hooping cough, croup, diarrhœa, dysentery, cholera, influenza, ague, remittent fever, typhus, erysipelas, quinsy, bronchitis, jaundice, and carbuncle. The districts of deaths were London, Devon, and Cornwall.

Out of the 139,318 cases thus chronicled as occurring from the above named diseases, the per centage of mortality in the different quarters, and estimating the gross mortality according to the season, without reference to particular years, ran as follows:

| | |
|---|---|
| In January, February and March .. | 25 per cent. |
| In April, May and June.. .. .. | 21 ,, |
| In July, August and September .. .. | 24 ,, |
| In October, November and December .. | 28 ,, |

Having learned thus much, I set about ascertaining, on the same large scale, whether the fatal diseases were in any way special to the seasons. The answer to this inquiry is to this effect:

Hooping cough, croup, small-pox, and bronchitis are most common to the first quarter. The per centage is:

| | 1st Quar. | 2nd Quar. | 3rd Quar. | 4th Quar. |
|---|---|---|---|---|
| Small pox .. | ..27,352 .. | 24,551 .. | 22,824 .. | 25,272 |
| Hooping cough | ..32,704 .. | 27,825 .. | 17,116 .. | 22,354 |
| Croup .. .. | ..27,523 .. | 25,100 .. | 19,919 .. | 27,456 |
| Bronchitis .. | ..36,793 .. | 20,301 .. | 10,327 .. | 32,570 |

Pneumonia, I believe, might very properly have been added here. In the second quarter, quinsy only stands a-head.—Thus:

| | 1st Quar. | 2nd Quar. | 3rd Quar. | 4th Quar. |
|---|---|---|---|---|
| Quinsy .. | ..21,762 .. | 30,596 .. | 21,231 .. | 26,410 |

In the third quarter, diarrhœa, dysentery and jaundice take the lead, in the following order:

| | 1st Quar. | 2nd Quar. | 3rd Quar. | 4th Quar. |
|---|---|---|---|---|
| Diarrhœa .. | ..10,196 .. | 10,717 .. | 58,519 .. | 20,567 |
| Dysentery .. | ..15,638 .. | 13,541 .. | 42,460 .. | 28,340 |
| Jaundice .. | ..24,877 .. | 24,030 .. | 26,967 .. | 24,109 |

In this third quarter, Asiatic cholera, when epidemic, assumes a greater mortality and prevalence than at any other season. Sporadic cases of cholera are, however, possibly more prevalent in the fourth quarter, during which influenza, ague, remittent fever, typhus, scarlet fever, measles, erysipelas, and carbuncle take the lead;

| | 1st Quar. | 2nd Quar. | 3rd Quar. | 4th Quar. |
|---|---|---|---|---|
| Influenza .. | ..23,539 .. | 12,171 .. | 4,502 .. | 59,784 |
| Ague .. .. | ..22,857 .. | 24,285 .. | 20,000 .. | 32,851 |
| Remittent fever | ..23,077 .. | 26,315 .. | 23,481 .. | 27,125 |
| Typhus .. | ..25,741 .. | 24,825 .. | 22,912 .. | 26,521 |
| Scarlet fever | ..20,809 .. | 18,978 .. | 26,234 .. | 33,976 |
| Measles .. | ..19,864 .. | 21,466 .. | 26,234 .. | 32,434 |
| Erysipelas .. | ..25,144 .. | 23,444 .. | 22,337 .. | 29,174 |
| Carbuncle .. | ..26,771 .. | 19,685 .. | 24,409 .. | 29,133 |

In a pathological as well as in a statistical point of view, these results are most interesting; for they prove, in a great measure, that diseases analogous in their general characters group themselves singularly together at special periods. Thus we see that, in the autumn quarter, there are grouped together those diseases which have for one of their essential symptoms an exudation from the intestinal surface, or that large abdominal viscera the liver. In the first quarter, the diseases of the respiratory system—croup, hooping cough, and bronchitis—stand forth prominently; while in the fourth quarter, a large family of diseases of the febrile or inflammatory order take the first position.

It is not by mere accident that these divisions occur: they are the effects of fixed, though nearly unknown physical or chemical laws.

It is worthy of special remark, that in the present quarter of the year—the fourth—the number of diseases which cause a prominent mortality is, as a general rule, greatest; and that next to it is the quarter commencing with the new year. During the third quarter of the present year, the mortality has been lower than is usual; a result due to the absence of any zymotic disease in a virulent form. As the cold of winter more decidedly sets in, we shall begin to see developed, almost of necessity, an increase of deaths from pulmonary diseases, and of low fever amongst the poor, if provisions become high in price and insufficient in quantity or quality.

In public practice, it is almost always to be observed that diarrhœa is a common symptom amongst the poor during intensely cold weather. In this form it does not, however, prove very fatal; and hence it stands low at that time in the mortality returns. It seems to occur simply from internal congestion, arising from long exposure of the external surface to cold; and there is no remedy for it that answers better than a good flannel under dress, and sufficient relays of food of a character to support the process of animal combustion. Except in the adoption of true sanitary reforms, there is no preventive of many of the zymotic diseases. It is not always possible even to get out of their way—for they are insidious visitants, and come when they are neither called nor expected. However, with some other diseases common to this and the next season, viz., diseases of the respiratory organs, and diarrhœa, much may be done in the way of prevention.

In regard to preventive measures, the multitude generally run into two opposite extremes—*i. e.*, they either take much more care of themselves than is required, or they do not take any care of themselves at all. They may be divided into two races, the Hardies and the Coddlers. In a word, both these classes set the simple and wise guiding genii of health, the instinctive feelings, at equal defiance. The man of the first class openly boasts his hardiness: "nothing hurts him." His father lived up to 90, and his mother to 99. Plenty of fresh air and no coddling is his decisive motto. If he caught himself clothed in under flannel, he would blush with very shame, as though every one could see through it; and he would consider it perfectly imbecile to have a little hot water with which to melt the ice that floats about his water-jug. As to his children, why should he make a fuss about them? The atmosphere may be at 32°, but to let them run out with bare legs and chests, is a bracing process; and their mottled state of skin, looking like an acute attack of purpura, is a proof of their hardiness and good health. This individual, however, feels all through that for the sake of a mere prejudice he is rebelling against a natural law. He is not comfortable. But to him the hydrostatics of the body, the chemistry of life, and even instinct are nothing—so he prepares himself and his children as sacrifices to pneumonia or bronchitis, and it is a perfect marvel if he does not at some time drive himself or them to the altar.

The antipode of this hardy mortal is the Coddler; the man who, on the slightest feeling of cold, gets into the fire and half roasts himself; who puts a weight of clothes on his back that would bear down Hercules; who thinks that constant relays of hot stimulating drinks are required to warm his stomach, and likes it all the better if his fingers and toes tingle again under the influence; who puts his feet in hot water for a mere sneezing fit; who half poisons himself with set formulæ of pills; who keeps his children in a close nursery throughout the whole of a frost; and who fancies that to be what he considers "cozy", is to be at once in a healthy and wise position. This man does himself more mischief than the former, for he soon becomes unable to meet the physical modifications of life, and succumbs easily to the first physiological accident arising from that exposure which he cannot always avoid.

If I were asked for a set of technical personal health rules, I should decline the task, though I were primed with all the medical learning since the era of old Esculapius. Technical rules on health are always hazards. If they are incorrect they are absolutely pernicious, and are so many nails driven before time into the coffin of him who follows them; if they are in some degree correct they are limited, and are too often mere shackles to the wearer. The golden health-rule is revealed to us all; it is a birthright. It bids us only to follow the dictates of the sensational spirit within us; to make the will in some measure the servant of this spirit, not its inexorable master. If a man is hungry or thirsty, let him eat till the instinct cries out, as it always does, "Enough." If he is neither hungry nor thirsty, let him abstain; for why should he do anything more till the appetite comes? He has no use for food until then, and it is more than a breach of economy to pour fluid into the full vessel. If he is cold, let him put on more clothes; and if the muscles or the mind fail, either from weariness or illness, let him go to bed and rest both.

Granting that the mountain will not come down to Mahomet, the Prophet must even go up to the mountain; granting that the seasons will not adapt themselves to the man, the man must adapt himself to the seasons. To this adaptation he was born, and he is by intuition endowed with the power of profiting by it. But in the course of his artificial culture of the will and the intellect, he ignores and opposes the natural dictator, and that original knowledge of health which was intended to make him his own physician. Unfortunately, too, in the present chaos of society, there are many who cannot obey the inner monitor even if they would. But for these obstacles I for one believe that half the diseases of our race might become as extinct as the dodo, and that such indefatigable vital philosophers as Dr. Farr might soon be obliged to find other spheres of labour than those which, by a lamentable necessity, they now so usefully and worthily occupy.

12, Hinde Street, Manchester Square,
December 12th, 1855.

